# Should Pregnant Women Consume Probiotics to Combat Endocrine-Disrupting Chemical-Induced Health Risks to Their Unborn Offspring?

**DOI:** 10.3390/biomedicines12081628

**Published:** 2024-07-23

**Authors:** Cheryl S. Rosenfeld

**Affiliations:** 1 Biomedical Sciences, University of Missouri, Columbia, MO 65211, USA; rosenfeldc@missouri.edu; 2 MU Institute for Data Science and Informatics, University of Missouri, Columbia, MO 65211, USA; 3 Department of Genetics Area Program, University of Missouri, Columbia, MO 65211, USA; 4 Department of Thompson Center for Autism and Neurobehavioral Disorders, University of Missouri, Columbia, MO 65211, USA

**Keywords:** bisphenol A, genistein, xenoestrogens, gut microbiota, microbiome, DOHaD, neurobehavioral disorders, gestation, in utero

## Abstract

Endocrine-disrupting chemicals (EDCs) have become so pervasive in our environment and daily lives that it is impossible to avoid contact with such compounds, including pregnant women seeking to minimize exposures to themselves and their unborn children. Developmental exposure of humans and rodent models to bisphenol A (BPA) and other EDCs is linked to increased anxiogenic behaviors, learning and memory deficits, and decreased socio-sexual behaviors. Prenatal exposure to BPA and other EDCs leads to longstanding and harmful effects on gut microbiota with reductions in beneficial bacteria, i.e., gut dysbiosis, and such microbial changes are linked to host changes in fecal metabolites, including those involved in carbohydrate metabolism and synthesis, and neurobehavioral alterations in adulthood, in particular, social and cognitive deficits. Gut dysbiosis is increasingly being recognized as a key driver of a myriad of diseases, ranging from metabolic, cardiovascular, reproductive, and neurobehavioral disorders via the gut-microbiome–brain axis. Thus, EDCs might induce indirect effects on physical and mental health by acting as microbiome-disrupting chemicals. Findings raise the important question as to whether pregnant women should consume a probiotic supplement to mitigate pernicious effects of EDCs, especially BPA, on themselves and their unborn offspring. Current studies investigating the effects of maternal probiotic supplementation on pregnant women’s health and that of their unborn offspring will be reviewed. Data will inform on the potential application of probiotic supplementation to reverse harmful effects of EDCs, especially BPA, in pregnant women unwittingly exposed to these compounds and striving to give their offspring the best start in life.

## 1. Introduction

Endocrine-disrupting chemicals (EDCs) include a broad range of natural and synthetic compounds that are present within industrial products (e.g., plastic containers, canned goods, water bottles, etc.), food items, and other daily-use household items. Importantly, these industrial chemicals are now produced in such voluminous amounts, with some being in billions of pounds per year, that they are now widely prevalent in terrestrial and aquatic environments, where they continue to bioaccumulate because of the inability to breakdown these manufactured substances. These chemicals are termed endocrine disruptors, as they can act to stimulate or suppress normal hormone responses in humans and a variety of animal species. EDCs induce their effects through a variety of mechanisms, including targeting hormone biosynthesis; suppressing release and transport of steroid hormones; binding steroid-hormone and non-steroid-hormone receptors in an agonistic or antagonistic manner, which prevents natural endogenous hormones and other factors from accessing their cognate receptors; and activating or suppressing endogenous steroid-hormone metabolism. While select EDCs might be natural chemicals, such as those found in plants, e.g., genistein, the majority of EDCs are manufactured chemicals used in the production of plastics and other household items [1]. Bisphenol A (BPA) has become one of the highest-volume and mass-produced petroleum-based chemicals that is used in the synthesis of a wide range of products, including plastics, food and beverage packaging materials, personal care products, and thermal paper receipts [2,3,4,5,6,7]. Its stability and pervasiveness have ensured continued exposure in the coming decades [7,8,9]. It is detectable in the urine of 93% of the US population [10], as well as in fetal plasma, placentas, and breast milk [11,12]. One study reported that BPA concentrations ranged from 0.3 to 18.9 ng/mL (median = 3.1 ng/mL) in maternal plasma, from 0.2 to 9.2 ng/mL (median = 2.3 ng/mL) in fetal plasma, and from 1.0 to 104.9 ng/g (median = 12.7 ng/g) in placental tissue [13]. In response to consumer demand, the US Food and Drug Administration (FDA) banned BPA in baby products, such as baby bottles and sippy cups, but this action fails to protect pregnant women and their unborn babies from being exposed to this chemical. The in utero period is the most critical, as this is time when organ systems of the fetus are being launched, and disruptions in their early formation can lead to longstanding health consequences. 

Early exposure to BPA and other EDCs, including ethinyl estradiol and genistein, alter, in a sex-dependent manner, the gut microbiota profile in rodent models [14,15,16,17,18,19,20]. Since our original studies, findings from other investigators have linked EDC exposure, including during the in utero period, and changes in the gut microbiota in humans, rodent models, frogs, crayfish, and zebrafish [21,22,23,24,25,26,27,28,29,30,31,32,33,34,35,36,37,38,39,40,41]. Thus, EDCs might also be considered “microbiome-disrupting chemicals”. The gut microbiome influences almost all host organ systems through a variety of mechanisms, detailed below. Changes in the gut microbiome, otherwise known as gut dysbiosis, can result in systemic host diseases, such as gastrointestinal, cardiovascular, reproductive, metabolic, and neurobehavioral disorders [42,43,44,45,46,47,48,49,50,51,52,53,54]. Until government policies are enacted across the world to prevent the manufacture and release of EDCs, alternative prevention and remediation strategies are needed, including in pregnant women seeking to protect their own health and to adapt measures to promote the lifelong health of their sons and daughters. Consumption of probiotics by pregnant mothers and those desiring to become pregnant is hypothesized to mitigate the effects of EDCs, especially BPA, on gut microbiota and thereby help prevent later diseases in their offspring. This review will explore the usage of maternal probiotic supplementation to combat pregnant women’s exposure to EDCs and alleviate EDC-induced pathological changes in their unborn sons and daughters, with an emphasis on neurobehavioral and metabolic disorders.

## 2. DOHaD Concept and Effects of Developmental Exposure to Endocrine-Disrupting Chemicals on Neurobehavioral Programming

The late Sir David Barker was one of the first to recognize the significance of the in utero period in shaping lifelong health [55,56,57]. His concept of fetal origin of adult disease (FOAD) has morphed into the accepted term of developmental origin of adult health and disease (DOHaD) to include the idea that extrinsic and intrinsic factors experienced during the periconception period for men and women and during pregnancy for women can lead to beneficial or adverse effects on their sons and daughters [58,59,60,61]. Human epidemiological and animal studies, including my research group’s own, have shown that fetal exposure to BPA and other EDCs dramatically increases the risk for a variety of diseases, including neurobehavioral disorders, such as autism spectrum disorders and autistic-like behaviors in rodent models [15,62,63,64,65,66,67,68,69,70,71,72,73,74,75,76,77]. BPA can act through estrogen receptor 1 and 2 (ESR1 and ESR2) and likely other steroidogenic and non-steroidogenic receptors to disrupt normal organizational–activational programming of the fetal brain [64,65,78]. However, EDCs can also affect microbiota that reside in our body, with the primary ones being in the gastrointestinal system (gut microbiota), as discussed below. 

## 3. Effects of Endocrine-Disrupting Chemicals on the Gut-Microbiome–Brain Axis

While the American Poet Walt Whitman may have been unaware about bacteria harbored within our body, his line “*I am large, I contain multitudes*” aptly applies, as the gastrointestinal system, uterus, vagina, seminal vesicles, oral cavity, skin, and other organs serve as a reservoir for a variety of bacteria with the gut overwhelmingly being the primary site [79,80,81,82,83,84,85,86]. More than 10,000 different bacteria species have been identified in the human body, and their collective genes dwarf ours at a ratio of 100:1 (3.3 million to 22,000). Over 90% of diseases, including cardiovascular, metabolic, immunological, reproductive, and neurobehavioral disorders, trace their origins back to disruptions in the gut microbiota, otherwise called gut dysbiosis [42,43,44,45,46,47,48,49,50,51,52,53,54]. 

**Figure 1:**
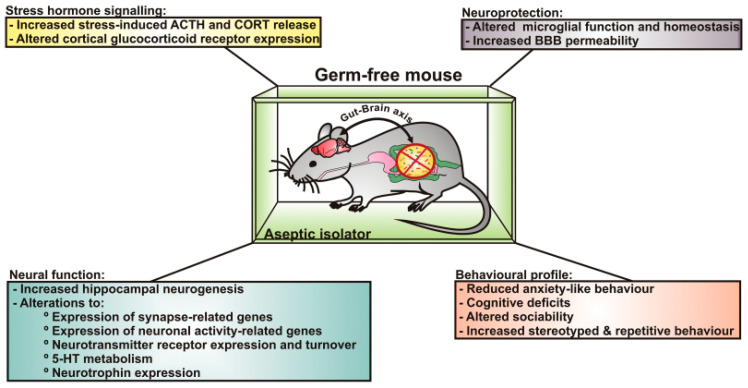
Hormonal and behavioral changes observed in germ-free (GF) mice that established the existence of a gut-microbiome–brain axis. GF mice must be raised in aseptic conditions to prevent exposure to microorganisms that might otherwise colonize the gut and other organ systems. The absence of gut bacteria results in profound changes in stress-hormone signaling pathways, morphological and gene-expression changes in the brain, altered responses to pathogenic organisms due to changes in microglial cells and increased blood–brain-barrier (BBB) permeability, and altered behavioral responses. This figure is from [94] and reproduced from permissions from Oxford University Press and Copyright Clearance Center, Inc.

The initial evidence for a gut-microbiota–brain axis or how changes in gut microbiota might lead to neurobehavioral diseases came from phenotypic alterations observed in germ-free (GF), axenic mice that lack gut microbiota. Such mice must be delivered via Caesarean section to avoid any potential transfer of maternal microbiota, and the offspring then have to be maintained under stringent and sterile conditions. Initial work established that restraint stress of adult GF mice led to increased production of adrenocorticotropic hormone and corticosterone (two commonly associated stress hormones) compared to specific-pathogen-free (SPF) controls [87]. However, provisioning GF mice with *Bifidobacterium infantis* alleviated the exaggerated hypothalamic–pituitary-axis (HPA) responses. Further, fecal microbial transfer from SPF to GF animals partially reversed the exaggerated hormonal responses but only if such intervention was performed early in life, suggesting that there is a critical postnatal window where microbes must colonize the gut for normal neural programming to occur. While this inaugural study did not assess behaviors in GF animals, the hormonal responses would suggest that GF mice would have increased anxiogenic behaviors. However, later work revealed GF animals were less anxious and more exploratory [88,89,90]. Moreover, the anxiolytic behavior was not impacted by SPF fecal transplantation [91]. GF mice, however, demonstrated cognitive deficits in nonspatial and working memory tasks [92]. A subsequent study supported these initial results, especially in males, and showed that GF mice avoided social situations [93]. Postweaning bacterial colonization abolished the cognitive but not social deficits, further evidence that there is a critical window for gut microbiota colonization and normal behavioral responses. Figure 1 illustrates select hormonal and neurobehavioral changes observed in GF mice.

The most-compelling evidence linking gut microbiota alterations and ASD-like behaviors in a mouse model was a study that used maternal immune activation (MIA) to induce ASD-like behaviors [95]. Treatment of pregnant mouse dams with the immunostimulant polyinosinic:polycytidylic acid causes offspring neural disturbances, which were attributed to disruptions in the gut-microbiome–brain axis, including resulting in gut dysbiosis, bacterial metabolite changes that increased the intestinal lining to become leaky. Importantly, treatment of these offspring with *Bacteroides fragilis* restored the intestinal barrier and gut microbiota residents, ameliorated the metabolome changes, and reversed the defects in communicative, stereotypic, anxiety-like, and sensorimotor behaviors otherwise observed in these mice. One metabolite that was restored to normal concentrations in the MIA offspring provisioned with this bacterium was 4-ethylphenylsulfate (4-EPS), which has been implicated as an autistic biomarker [96]. This key study provided the most robust and causative evidence that gut dysbiosis induces a cascade of host pathophysiological changes leading to ASD-like behavioral disturbances. However, probiotic treatment with a single bacterium in this animal model mitigated the pathologic changes in the gut, mitigated bacterial metabolic disruptions, and reversed the neurobehavioral abnormalities.

**Figure 2:**
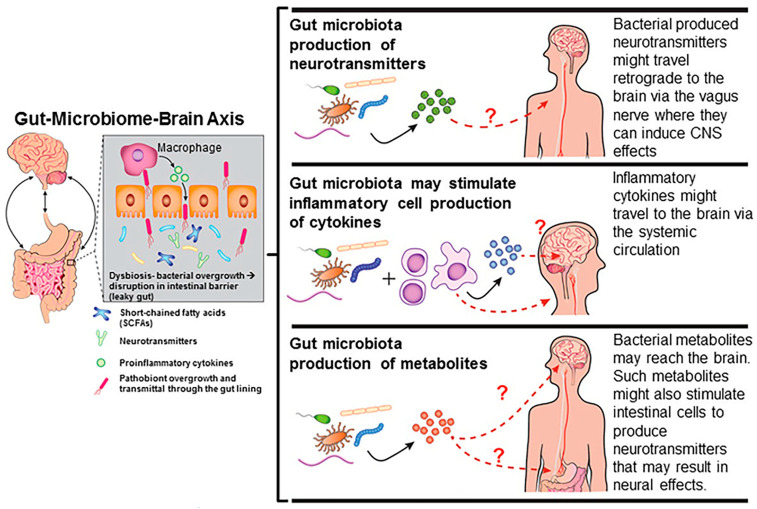
Select mechanisms by which the gut microbiome may influence the brain and other organs, reproduced from [19]. This is an open-access article distributed under the terms of the Creative Commons Attribution License (CC BY). The use, distribution, or reproduction in other forums is permitted, provided the original author(s) or licensor are credited and that the original publication in this journal is cited, in accordance with accepted academic practice.

**Figure 3:**
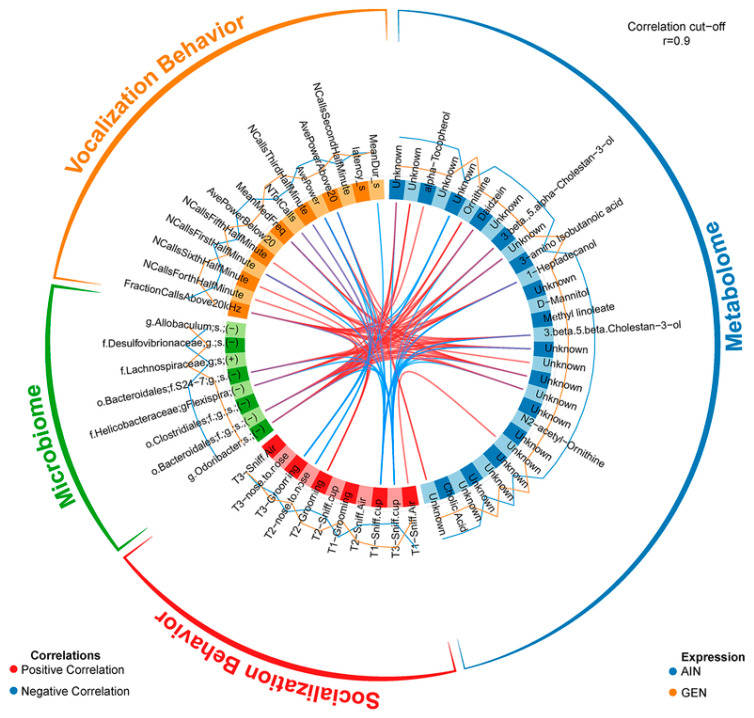
A mixOmics analysis approach to integrate, in this case, developmental exposure to genistein (GEN) vs. AIN (phytoestrogen-free diet) on gut microbiota changes with fecal metabolite and neurobehavioral changes in vocalizations and social behaviors. This integrative approach is one of the few that permits correlations between multiple omics approaches and phenotypic changes. Positive correlations are shown by the red lines linking specific categories together, whereas inverse or negative correlations between two categories are represented in blue. The correlation strength was set to 0.9, which is quite stringent [17]. This figure was reproduced with permissions from Copyright Clearance Center, Inc.

Gut microbiota influence metabolism and behavior through the gut-microbiome–brain axis [14,15,16,17,18,46,97,98]. These effects can be due to alterations in bacterial-derived neurotransmitters, short-chain fatty acids (SCFAs) that result in epigenetic and genetic changes in neuronal cells, inciting inflammatory responses, and altering the blood–brain barrier (Figure 2) [18,46]. Thus, EDCs might induce indirect effects on physical and mental health by acting as “*microbiome-disrupting chemicals*”. My research group was one of the first to show that developmental exposure to BPA and other EDCs, including ethinyl estradiol and genistein, alter, in a sex-dependent manner, the gut microbiota profile in California mice (*Peromyscus californicus*) [14,15,16,17,18,19,20,98]. With mixOmics analysis, my group was able to link gut microbiota changes with alterations in fecal metabolites and behavioral patterns (Figure 3). 

Other reports have correlated EDC exposure with gut microbiome changes and potential host diseases, including in humans and changes in the gut microbiota in humans, rodent models, frogs, crayfish, and zebrafish [21,22,23,24,25,26,27,28,29,30,31,32,33,34,35,36,37,38,39,40,41]. In a pilot study with mother–infant pairs from sub-Saharan Africa, correlations were detected between chemical exposure, including tricetin and vulgaxanthin, and changes in the infant gut microbiome [21]. Findings from another human epidemiological study suggest that changes in infant gut microbial communities might serve as biomarkers for pre- and postnatal exposure to EDCs, including BPA and phthalates [38]. Male zebrafish (*Danio rerio*) treated with estradiol (E2) or BPA for weeks showed increased expression of vitellogenin in the liver and changes in intestinal microbial communities with an increased abundance of phylum CKC4 [32]. 

Bisphenol S (BPS) is the analogue of BPA and has been proposed as a safer alternative. However, mice exposed for 154 days to BPS exhibit gut dysbiosis, obesity, hepatic lipid accumulation, intestinal lesions, and dyslipidemia, and investigators found a significant correlation between BPS-induced gut microbiota changes and indicators of host health [24]. Increased concentration of BPS in the water results in intestinal dysbiosis and accumulation of lipids and damage in the hepatopancreas of freshwater crayfish (*Procambarus clarkia*) [34].

Di-2-ethylhexyl phthalate (DEHP) is a plasticizer present in polyvinyl chloride, medical equipment, and food packaging. Zebrafish exposed to DEHP through diet exhibit gut dysbiosis, including altered microbial diversity and functionality [40]. Male and female zebrafish provisioned daily with DEHP for two months had altered gut microbiota populations and corresponding changes in metabolites regulating the gastrointestinal system and T helper cells, suggesting that DEHP can impact the gut-microbiome–immune axis [41]. Pregnant mouse dams treated with DEHP for 10 days and euthanized 21 days after their last dose showed alterations in cecal microbiota [25]. While no changes in fertility or birth outcomes occurred in the DEHP-treated dams, changes in gut flora in these females may have predisposed her offspring to later diseases, which was not assessed. Male mice treated early in life with doxycycline, which acts both as an antibiotic and anti-testosterone agent, had gut microbiota alterations, including reductions in overall diversity and compositional changes, along with changes affecting testes transcripts for genes associated with Leydig cells (*Cyp11a1*, *Cyp17a1*, and *17β-HSD*) and spermatogenic cells (*Grfal*, *Plzf*, and *Stra8*) [29].

Octylphenol (OP) is another widely distributed EDC present in diverse terrestrial and aquatic environments. Exposure of *Rana chensinensis* tadpoles to OP in the water affected transcripts related to fat digestion and absorption, altering the structure and composition of intestinal microbiota, with a decreased ratio of Firmicutes/Bacteroidetes that was associated with disrupted lipid metabolism [31]. Mice exposed to the EDC carbendazim for 28 days showed increased hepatic lipid accumulation and triglyceride levels, elevations in serum cholesterol, and high-density and low-density lipoproteins with corresponding elevations in gene expression related to lipogenesis and triglyceride synthesis in liver and fat tissue [30]. Inflammatory markers IL-1β and IL-6 were increased in the serum, and a significant reduction in richness and diversity of cecal microbiota was evident in treated mice. The relative abundances of Firmicutes, Proteobacteria, and Actinobacteria were elevated, but that of Bacteroidetes decreased in the gut microbiota of carbendazim-exposed mice.

**Figure 4:**
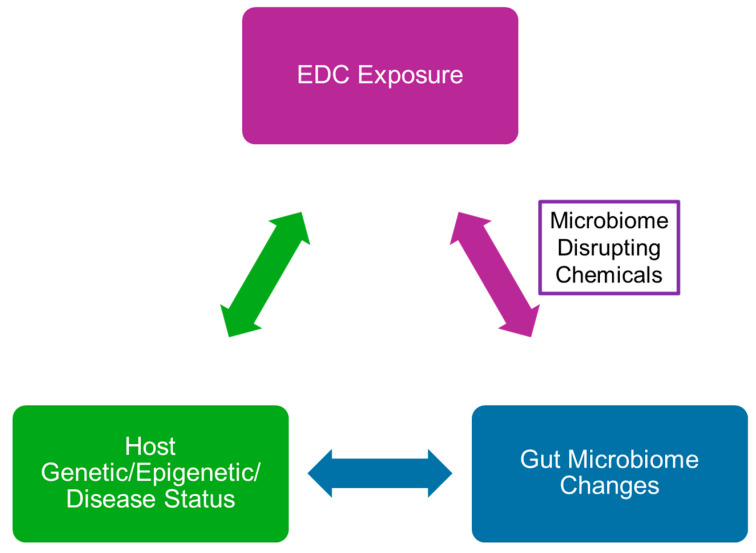
The triad relationship between developmental exposure to EDCs, such as BPA, gut-microbiome changes, and host genetic/epigenetic/phenotypic status. If individuals cannot eliminate exposure to EDCs and it is unrealistic to reverse direct effects of EDCs on our genetic, epigenetic, and disease status, then EDCs acting as microbiome-disrupting chemicals might provide an avenue to intervene and abate the harmful effects of EDCs.

**Figure 5:**
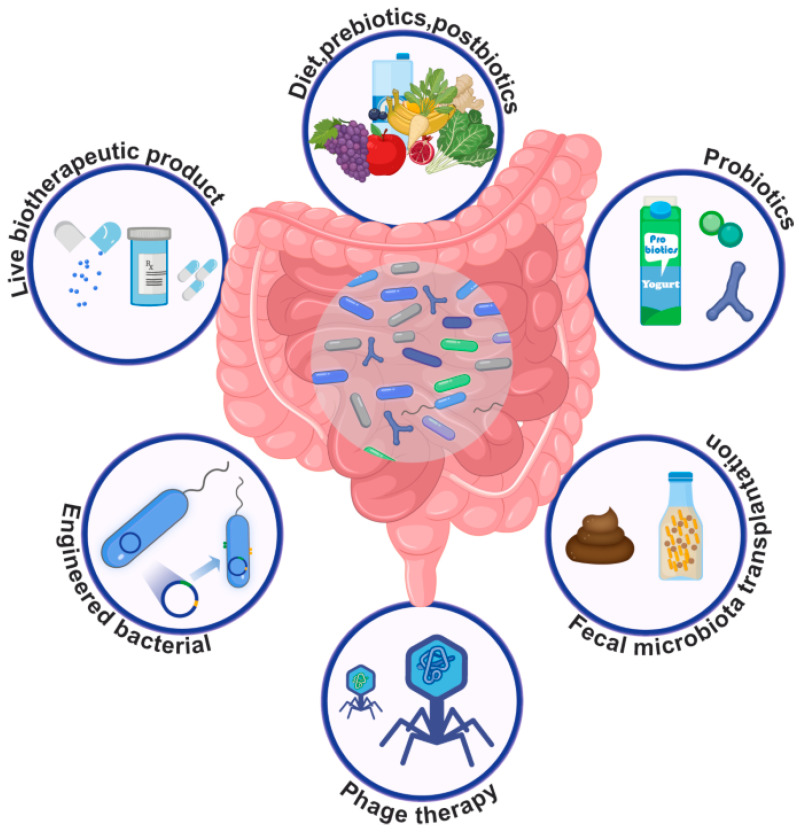
Intervention mechanisms to modify gut microbiota and/or their products. In so doing, such approaches might be used to prevent/treat human diseases, including those originating due to early exposure to EDCs, such as BPA. This figure is from [100]. This publication is an open-access article distributed under the terms of the Creative Commons (CC BY) license, which permits unrestricted use, distribution, and reproduction in any medium, provided the original work is properly cited.

Collectively, results suggest that a triad relationship exists with exposure to EDCs leading to direct effects on host health that, in turn, can affect gut bacterial populations, but EDCs might also lead to direct effects on the gut microbiota that result in host diseases (Figure 4). Potential reversal of EDC effects on gut microbiota might thus partially to fully mitigate the effects of developmental exposure to EDCs on our health. Mechanisms by which gut-microbiota–host interactions can be altered are summarized in Figure 5. These include supplementation of prebiotics that promote the growth of certain gut bacteria over others; probiotic supplementation to encourage colonization of the gastrointestinal system with beneficial bacteria; postbiotic administration, where bacterial products, such as SCFAs, are provided to influence host responses; fecal microbial transfer from a healthy donor to a recipient who might have gut dysbiosis, leading to pathological changes within the individual; phage therapy to target pathogenic bacteria (pathobionts); bacteria engineered to have removal of harmful products (e.g., lipopolysaccharide) or inclusion of genes to encode for products favorable to the host; and, lastly, live biotherapeutic products (LBPs) or microbiome-based therapeutics that may restore eubiosis. The US Food and Drug Administration (FDA) has defined LBPs as biological products that “*(1) contain live organisms, such as bacteria; (2) are applicable to the treatment, prevention or cure of a disease, and (3) are not a vaccine*” [99]. For instance, the FDA approved single-dose administration of live-jslm (RBL) bacteria from human feces to treat *Clostridium difficile* infections (rCDIs) in adults [99]. LBPs might prove to be innovative approaches to treat specific diseases, but these therapies are in their infancy of understanding. Of the mechanisms listed above to modify the gut microbiome, the focus of most studies has been on probiotic supplementation. Yet, questions remain on the efficacy of this approach in alleviating host disease due to intrinsic or extrinsic factors, namely EDC exposure. 

## 4. Effects of Probiotics on Health of Pregnant Women

Pregnant women should be empowered to accomplish everything they can to protect their own health and promote the lifelong health of their unborn sons and daughters, and they are craving answers as to what they can accomplish in the face of not being able to avoid all EDC exposure or contact with other environmental chemicals. Probiotic/synbiotic (mixture of probiotics and prebiotics) supplementation during pregnancy in general leads to improved health outcomes, including reduction in the risk for metabolic disorders, such as gestational diabetes mellitus (GDM), hypertension, depression, anxiety, pre-eclampsia (PE), and other gestational disorders [101,102,103,104,105,106,107,108,109,110,111,112,113,114,115,116].

A meta-analysis representing 4356 pregnant women found that probiotic supplementation correlated with decreased risk for atopic eczema, increased gestational age, and reduced risk for death and necrotizing enterocolitis (NEC) [101]. A network meta-analysis study representing 32 studies considered the effects of maternal probiotic supplementation with the following bacterial combinations: *Lactobacillus*, *Lacticaseibacillus rhamnosus,* and *Bifidobacterium* (LRB); *Lactobacillus acidophilus* and *Bifidobacterium* (LABB); *Lactobacillus acidophilus*, *Lacticaseibacillus casei*, and *Bifidobacterium bifidum* (LLB); multi-combination of four probiotics (MP1); and multi-combination of six or more probiotics (MP2). LLB, MP1, and MP2 all contain LABB [103]. The findings revealed that the multi-combinations of probiotic strains, particularly those containing LABB, are more effective than a single probiotic strain in regulating glycolipid metabolism, inflammation, and oxidative stress in pregnant women on these supplements [103]. 

A meta-analysis with 2213 participants reported that maternal probiotic supplementation greatly reduced the risk ratio for GDM, reduced fasting serum glucose and insulin concentrations, and decreased the homeostasis model assessment of insulin resistance (HOMA-IR) [104]. Results suggest that probiotic supplementation may lead to an improvement in glycemic control and reduction in GDM incidence in pregnant women. Another meta-analysis with 1119 study participants revealed that maternal probiotic supplementation lowered maternal serum glucose, insulin levels, and HOMA-IR indices but only in pregnant women with gestational diabetes mellitus [102]. Synbiotics also lowered serum insulin levels and HOMA-IR index. Taken together, these findings suggest that probiotics might be useful to improve glucose metabolism in pregnant women with GDM. Collective analyses of 54 randomized controlled trials with 9443 pregnant women suggest that consumption of probiotics by pregnant women might reduce cardiometabolic risk and subsequently help prevent GDM [106]. Other meta-analyses and reviews of cohort studies provide robust evidence that consumption of probiotics during pregnancy is associated with lower risk for GDM, metabolic syndrome, inflammation, and oxidative stress [107,108,109,110].

A meta-analysis of 16 randomized controlled trials with 946 pregnant women found that consumption of probiotics was associated with reduced anxiety and depression that corresponded with positive gut microbiota changes in these individuals [112]. A study with 128 pregnant women with mild PE and gestational ages exceeding 24 weeks were provided either a symbiotic capsule or placebo [113]. Those who received the synbiotic capsule had lower mean systolic and diastolic blood pressures, and the incidences of progression to severe PE, proteinuria, and mean serum creatinine level significantly declined. This pilot study suggests that synbiotics might be useful in preventing pregnancy complications. An observational study with 70,149 singleton pregnancies resulting in live-born babies from the Norwegian Mother and Child Cohort Study reported an association between probiotic milk consumption during pregnancy and reductions in the incidence of PE and preterm delivery [115]. An earlier study with this same cohort population also found that regular consumption of milk-based probiotics was linked with reduced risk for PE in primiparous women [116].

In pregnant mice, PE-like signs were induced by injection of N-Nitro-L-arginine methylester (a nonselective inhibitor of nitric oxide synthetases, NOS) intraperitoneally from gestational day 11 through 18, which results in increased systolic blood pressure and proteinuria. However, supplementation with the bacterium *Akkermansia muciniphila* alleviates these PE-like signs [114]. Pregnant female rats who underwent surgery to reduce uterine blood perfusion, as a model of PE, and were then provided a probiotic had reductions in serum endotoxin, improvements in metabolic excretion, increased colonization by beneficial bacteria in the intestine, improved vascular endothelial function, reduced blood pressure, and decreased systemic inflammatory responses relative to pregnant females with PE-like signs that did not receive this treatment [117]. 

## 5. Effects of Maternal Probiotics on Offspring Health

Animal model studies provide support for the usage of probiotics prior to pre-birth to protect against metabolic syndrome originating during the in utero period [118]. Increasing evidence suggests that probiotics and nutrient supplements during the preconception trough postnatal period might be strong determinants in shaping offspring health [119]. Consumption of probiotics by pregnant women may also reduce the risk for fetal growth restriction (FGR); allergies/asthma in their children, promoting offspring neurodevelopment; and later anxiety-like behaviors and metabolic diseases in male and female rodent offspring, including those born to obese dams [120,121,122,123,124]. These studies are reviewed below. Materan probiotic supplementation may have immediate in utero effects, as this treatment appears to relieve FGR-induced inflammation and improve fetal growth [122]. A comprehensive review and randomized clinical trials examining whether the consumption of probiotics by pregnant women reduced the later incident of allergies in their children concluded that this therapy might confer some protection, especially in children at risk of developing allergies [120]. 

An earlier meta-analyses representing 1223 pregnant women provided the individuals with either a probiotic or placebo treatment from 36 weeks of gestation [121]. Their infants were then provided the same treatment, along with prebiotic galacto-oligosaccharides for 6 months. At six months of age, infants who received the synbiotic had improved immune system function, as evidenced by enhanced vaccine antibody response, immune defense in the gastrointestinal system, and resistance to respiratory infections. While infants receiving a synbiotic did not show decreased allergies at two years of age, they had reduced incidence of eczema and other IgE-associated diseases at this age. However, by five years of age, no differences in atopic eczema, allergic rhinitis, and asthma were evident between the two groups.

Pregnant mice provided a high-fat diet have reduced gut short-chain-fatty-acid levels, increased markers of peripheral inflammation, and reductions in neuroactive metabolites in milk provided to their offspring [124]. Offspring born to these obese dams exhibited increased anxiogenic behaviors that are alleviated by maternal perinatal probiotic treatment. This supplementation during the perinatal period also increased gut butyrate and brain lactate levels and resulted in enhanced expression of neurogenic transcripts, such as brain-derived neurotrophic factor (*Bdnf*) in the offspring. These findings have important clinical ramifications, as they suggest that maternal probiotic supplementation can reverse harmful effects of maternal obesity on offspring brain development by increasing neuroplasticity, improving gut–liver–brain metabolome profiles, and improving resilience to emotional disturbances.

While this rodent study and human cohort studies examining the effects of maternal probiotic supplementation on offspring outcomes have shown beneficial effects, studies to date have not considered the potential of maternal probiotic supplementation to overcome health risks posed to themselves and their unborn fetuses following EDC exposure. Instead, the ability of probiotic supplementation to reverse diseases originating due to EDC exposure in adults of mice and zebrafish will be examined.

## 6. Usage of Probiotics to Neutralize Effects of Endocrine-Disrupting Chemicals

Evidence to date that probiotic supplementation in adult mice, rats, and zebrafish can mitigate EDC-induced disorders will be considered. Adult male mice were provisioned with two probiotic strains (*Lactobacillus rhamnosus* and *L. plantarum*) that had demonstrated in vitro to have BPA-removal properties, which were evaluated in male mice exposed to BPA [125]. BPA exposure alone resulted in suppression of vitamin-D metabolism and reduction in bile acid levels that correlated with increased abundance of *Faecalibaculum* and decreased abundance of Lachnospiraceae_NK4A136_group and *Ligilactobacillus*. However, co-treatment with the two probiotics improved the intestinal barrier function, reduced oxidative stress, and restored the balance of short-chain fatty acids (SCFAs). Male mice exposed to BPA had testicular abnormalities, including changes in the seminiferous tubules and arrested spermiogenesis, but such effects were mitigated in those concurrently treated with the probiotic mixture. The two-probiotic mixture enhanced testosterone-driven increases in spermatogonial stem cells and subsequent stages of spermatogenesis. Transcriptomic changes were noted in the testes of mice that were provided probiotic supplementation along with BPA exposure, which included improvements to fatty-acid degradation and peroxisome pathways. The combined results suggest gut microbiota can influence spermatogenesis and probiotics might be used to alleviate testicular changes due to BPA exposure. 

In adult rats, probiotic supplementation (*Saccharomyces boulardii* and *Lactobacillus rhamnosus* and *L. plantarum LP 6595* and *Lactobacillus plantarum HEAL9*) mitigated the effects of co-exposure to the EDC mixture of BPA, DEHP, and dibutyl phthalate (DBP) for 28 days on the pancreas, including improving redox status and potential development of type 2 diabetes mellitus [126]. Zebrafish that were provided the probiotic mixture (SLAb51) had reversal of the harmful effects of BPA exposure for 28 days on the microbiome–liver–brain axis and male and female reproductive systems [127]. 

## 7. Conclusions and Future Directions

My research group’s original studies using rodent models suggested that developmental exposure to EDCs, including BPA, genistein, and ethinyl estradiol, can function as microbiome-disrupting chemicals [14,15,16,17,18,19,20,98], which has now been supported in other rodent models, zebrafish, tadpoles, crayfish, and, notably, humans [21,22,23,24,25,26,27,28,29,30,31,32,33,34,35,36,37,38,39,40,41]. It is also clear that other EDCs, including BPS, tricetin, vulgaxanthin, phthalates, OP, and carbendazim, function as microbiome-disrupting chemicals. Developmental exposure to EDCs leads to sex-dependent differences in gut microbiota changes. My research group, and others, have also linked such EDC-induced microbiota changes and host diseases, including neurobehavioral and metabolic disorders. 

Maternal supplementation with probiotics might confer protection against EDC exposure and later health risks in the mother’s offspring. Probiotic supplementation might directly impact the pregnant woman’s gut, vaginal, and other microbiomes that she passes onto her offspring. These micro-organisms might also reduce absorption of EDCs in the maternal intestinal system and thereby reduce total exposure to the pregnant woman and transference of EDCs to the fetus via the placenta. Colonization of the maternal intestinal system with probiotics might also increase the production of beneficial bacterial products, including SCFAs, that transit across the placenta to the fetus and induce beneficial epigenetic and genetic changes in the host that override effects induced by developmental exposure to EDCs. In this aspect, postbiotic supplementation might also be considered to combat early exposure to EDCs, but further work is needed to determine which bacterial factors are beneficial to maternal and offspring health. Thus, current research studies first need to focus on whether maternal probiotic supplementation is advisable to abate EDC alterations in pregnant women’s unborn sons and daughters.

Most studies suggest that probiotic supplementation during pregnancy may improve maternal health, including lowering risk for depression, other mental health disorders, GDM, hypertension, PE, and other gestational disorders [101,102,103,104,105,106,107,108,109,110,111,112,113,114,115,116]. Maternal probiotic supplementation may improve offspring health outcomes, which range from improved fetal growth and immune status, lowered risk for allergies and eczema, to promoted neurodevelopment and resistance to emotional disorders due to maternal obesity, as observed in the rodent model study [120,121,122,123,124]. 

While no study to date has directly tested whether maternal probiotic supplementation can reverse harmful effects caused by EDC exposure, select studies have been performed in adult mice, rats, and zebrafish [125,126,127]. The collective findings from these studies suggest that provisioning of probiotics alleviates reproductive, metabolic, and gut microbiota changes due to EDC exposure. 

Findings provide staunch support for the usage of maternal probiotic supplementation to combat EDC exposure to pregnant women and their unborn offspring. It is during this critical window of time that fetal organ systems undergo initial formation and programming and can be sculpted for better or worse by extrinsic factors the mother is exposed to and consumes. Such studies also need to consider whether both sexes respond equally to maternal probiotic supplementation to overcome EDC exposure. Probiotic supplementation by pregnant mothers might need to be customized to accommodate whether she is carrying a son or daughter. Maternal probiotic supplementation should be evaluated in combination with a variety of EDCs to determine whether this strategy can be used to mitigate a wide assortment of EDC exposures. Other intervention methods to modify the gut microbiota or bacterial products, as shown in Figure 5, might also be considered alone or in combination as to their beneficial aspects in reversing EDC effects. The ability of such compounds to function as microbiome-disrupting chemicals can be leveraged to develop alternative strategies to combat developmental exposure to EDCs, in particular, maternal probiotic supplementation.
